# Small nucleolar RNA Snora73 promotes psoriasis progression by sponging miR-3074-5p and regulating PBX1 expression

**DOI:** 10.1007/s10142-024-01300-7

**Published:** 2024-01-19

**Authors:** Lihua Zhang, Hui Guo, Xiaoguang Zhang, Ling Wang, Feng Wei, Yike Zhao, Bo Wang, Yibo Meng, Yanling Li

**Affiliations:** 1 Department of Dermatology, Clinical Medical Research Center of Dermatology and Venereal Disease in Hebei Province, The Second Hospital of Hebei Medical University, Shijiazhuang, 050000 China; 2 Construction Unit of the Sub-Center of the National Center for Clinical Medical Research On Skin and Immunological Diseases, The Second Hospital of Hebei Medical University, Shijiazhuang, 050000 China; 3 Key Laboratory of Infection and Immunity of CAS, CAS Center for Excellence in Biomacromolecules, Institute of Biophysics, Chinese Academy of Sciences, Beijing, 100101 China

**Keywords:** Psoriasis, snoRNA, Snora73, miR-3074-5p, PBX1

## Abstract

Chronic psoriasis is a kind of immune-mediated skin illness and the underlying molecular mechanisms of pathogenesis remain incompletely understood. Here, we used small RNA microarray assays to scan the differential expressed RNAs in psoriasis patient samples. The downstream miRNAs and its targets were predicted using bioinformatics analysis from online bases and confirmed using fluorescence in situ hybridization and dual‑luciferase report gene assay. Cell ability of proliferation and migration were detected using CCK-8 and transwell assays. The results showed that a new snoRNA Snora73 was upregulated in psoriasis patient samples. Overexpression of Snora73 significantly increased psoriasis cells viability and migration, while knockdown of Snora73 got the opposite results. Mechanistically, our results showed that Snora73 acted as a sponge for miR-3074-5p and PBX1 is a direct target of miR-3074-5p in psoriasis cells. Furthermore, miR-3074-5p suppressed psoriasis cell proliferation and migration, while PBX1 promoted cell proliferation and migration in psoriasis. Collectively, these findings reveal a crucial role of Snora73 in progression of psoriasis through miR-3074-5p/PBX1 signaling pathway and suggest a potential therapeutic strategy.

**Supplementary Information:**

The online version contains supplementary material available at 10.1007/s10142-024-01300-7.

## Introduction

Psoriasis is common and inflammatory, which impacts roughly 2% of the ordinary populace (Dopytalska et al. 2021; Hindelang et al. 2022). The current therapy methods for psoriasis contain ultraviolet A photochemotherapy, vitamin D3 derivatives, retinoids, steroids, immunosuppressants, and biological therapeutics (Le and Torres 2022). However, the pathogenetic mechanism of psoriasis remains unclear.

Small nucleolar RNAs (snoRNAs) are usually considered as a type of non-coding RNAs with conserved functions in all known eukaryotes, which could conduct the modification of ribosomal RNA (Bratkovic et al. 2020; van Ingen et al. 2022). The expression of snoRNAs is stable and detectable in cancer patients’ blood plasma, serum, and urine, indicating a potential diagnosis marker role of snoRNAs (Fitz et al. 2021; Liang et al. 2019). SNORA73, also known as snoRNA U17, was found to inhibit hypoxia-upregulated mitochondrial movement regulator (HUMMR) during cholesterol trafficking (Jinn et al. 2015). A study showed that the absence of Snora73 reprograms cellular metabolism to prevent steatohepatitis and lipid-induced oxidative stress (Sletten et al. 2021). SNORA73 was one of the prognostic signatures of 14 snoRNAs for acute myeloid leukemia (Huang et al. 2022). However, the biological role of Snora73 in psoriasis cell dysfunction remains unclear.

MicroRNAs (miRNAs) are endogenous noncoding RNAs with about 22 nucleotides in length, which are highly conserved and participates in biological process through negatively regulated target mRNA expression, and has been reported to involved in psoriasis pathology (Wang et al. 2023; Wu et al. 2018; Zhou et al. 2022), which may be potential biomarkers or candidates for psoriasis therapy (Yan et al. 2022). MicroRNA-17-92 cluster promotes the proliferation and the chemokine production of keratinocytes in psoriasis (Zhang et al. 2018). MiR-223 regulates proliferation and apoptosis of IL-22-stimulated HaCaT human keratinocyte cell lines via the PTEN/Akt pathway (Wang et al. 2019). Research revealed that miR-3074-5p could be negatively regulated by a lncRNA Eif4g2 in mouse β-cells (Wang et al. 2020). These reports indicate that miRNAs maybe a molecular target for psoriasis therapy.

Pre-B-cell leukemia homeobox transcription factor 1 (PBX1) has been associated with important developmental programs (Veiga et al. 2021). PBX1 plays critical roles during embryogenesis, organogenesis, development, and differentiation. PBX1 participated in estrogen mediated BCa progression and chemo-resistance through binding and activating estrogen receptors (Zhao et al. 2022). However, the molecular mechanisms of PBX1 in psoriasis remains elusive. In this paper, we found high expression of Snora73 in psoriasis and serving as a sponge of miR-3074-5p. Also, we validated PBX1 was a miR-3074-5p target, which maybe a promising therapeutic target for psoriasis.

## Material and methods

### Samples

Skin biopsies and plasma were collected from 20 patients with psoriasis at the Department of Dermatology of The Second Hospital of Hebei Medical University. Inclusion and exclusion criteria for psoriasis are as follows: (1) aged 18 years or older, (2) with moderate to severe plaque psoriasis (Psoriasis Area and Severity Index (PASI) score ≥ 12, (3) ≥ 10% body surface area affected by psoriasis, and (4) Investigator’s Global Assessment (IGA) score ≥ 3 on a five point scale (Reich et al. 2021). Written informed consent was obtained from those patients. The current research received approvals from the Research Ethics Committee of The Second Hospital of Hebei Medical University (2023-R224). The used psoriasis patient’s information was shown in Supplementary Table 1.

### Cell culture and treatment

Cells of human adult low-calcium high-temperature (HaCaT) were supplied by the Shanghai Institute of Cell Biology, Chinese Academy of Sciences (Shanghai, China). Those cells were cultured in DMEM (Gibco, NY, USA) containing 10% FBS, 100 units/ml penicillin, and 100 µg/ml streptomycin at the temperature of 37° with 5% of CO_2_. The cells were inserted into 50 ng/ml IL-17A (Cat# P00044, Solarbio, Beijing, China) for 24 h. Then, the cells were fostered into keratinocyte‑induced HaCaT cells, which is a model of psoriasis in vitro.

### Fluorescence in situ hybridization (FISH)

An online tool of Biosearch Technologies (https://www.biosearchtech.com/) has devised Snora73 probes (5′-TGTCCACAGGACTCAGAAGCT-3′). Alexa Fluor 594-conjugated probes were supplied by Invitrogen. The cell slides were placed at the bottom of a six-well cell culture plate, and cells were seeded at a density of 1 × 10^5^/well. Then, cells were fixed with 4% paraformaldehyde at room temperature for 10 min, and then the cells were washed with 1 × PBS buffer for three times. Next, we permeabilize the cells with permeabilization solution (PBS containing 0.5% Triton X-100) at 4 °C for 20 min, followed by washing with 1 × PBS solution for three times. The pre-hybridization solution was blocked at 37 °C for 30 min, and the probe hybridization solution was added at 37 °C for overnight hybridization. Confocal microscopy (FV1200, Olympus) was used to visualize the samples.

### Cell transfection

miR-3074-5p inhibitor (5′-ACUGGCUCAGUUCAGCAGGAAC-3′) and Inhibitor control oligos (5′-CAGUACUUUUGUGUAGUACAA-3′) were supplied by Sangon Biotech Co., Ltd. (Shanghai, China). miR-3074-5p simulated (sense: 5′-GUUCCUGCUGAACUGAGCCAG-3′; antisense: 5′-CUGGCUCAGUUCAGCAGGAAC-3′), and mimic-NC (sense: 5′-UUGUACUACACAAAAGUACUG-3′, antisense: 5′-CAGUACUUUUGUGUAGUACAAA-3′) were purchased from Ribo Bio (Guangzhou, China). A total of 100 ng miRNAs of miR-3074-5p mimic, we transfected miR-3074-5p inhibitor and negative control separately into cells by means of Lipofectamine 2000 (Cat# 11668027, Thermo Fisher Scientific, Inc.). A total of 1.0 µg overexpression plasmid of Snora73 or PBX1 (pcDNA3.1) and empty control (vector) were transfected into the indicated cells using Lipofectamine 2000 (Cat# 11668027, Thermo Fisher Scientific, Inc.). It cost 6 h to transfect that substrate and which was then eliminated. Subsequently, the fresh medium containing 50 ng/ml IL-17A was added for 24 h.

### Cell viability assay

Briefly, we inserted the keratinocyte‑induced HaCaT cells (1.0 × 10^4^ cells/well) into 96-well plates and cultured them for 0, 1, 2, and 3 days. Ten microliters of CCK-8 solution (Cat# C0038, Beyotime, Shanghai, China) was added into per well. Then, it cost 2 h to foster those wells at the temperature of 37° with 5% CO_2_. At 450 nm in height, the absorbance determined via the plate reader (Bio-Rad, CA, USA).

### RNA isolation and qRT-PCR

TRIzol reagent (Cat# 15596026, Invitrogen, CA, USA) was adopted, with the objective to extracting the total RNAs from HaCaT cells or skin tissues. M-MLV reverse transcriptase (Cat# M1701, Promega Corporation, Madison, WI, USA) was used to reversely transcribe RNAs to cDNA. qRT-PCR was implemented by means of SYBR detection kit (Cat# RR420L, Takara, Tokyo, Japan) on a Real-Time PCR Detection System. 2-ΔΔCT approach was adopted, with the intention of analyzing mRNA levels of target genes. The used primer pairs are shown in Supplementary Table 2. The reaction conditions are as follows: holding stage, 95 °C, 3 min; cycling stage of 40, 95 °C, 15 s; 60 °C, 30 s; 72 °C, 30 s. 18sRNA (for snoRNAs and miRNAs assay) and GAPDH (for PBX1 assay) functioned as endogenous control. Experiments were performed in triplicate.

### Nuclear-cytoplasmic separation assay

Cytoplasmic and nuclear RNA was extracted using cytoplasmic/nuclear fractionation kit (Cat# NGB-21000, Norgen Biotek). Briefly, HaCaT cells were suspended and lysed with cell fraction buffer and then centrifuged at low speed to separate the nuclear fraction from the cytoplasmic fraction. Subsequently, the cytoplasmic fraction was carefully aspirated away from the nuclear pellet, and the cell disruption buffer was added to the nuclear pellet. *Snora73* cell distribution among HaCat cells measured via qRT-PCR. U6 and GAPDH were markers in nuclear and cytoplasmic.

### Arrystar small RNA expression array

Human psoriasis and normal plasma samples were collected for small RNA expression assay. RNA was extracted using TRIzol Reagent, dealt with DNase, and then depurated with RNeasy mini spin columns. The samples were quantitative by NanoDrop ND-1000 spectrophotometer. Besides, RNA integrity was detected using Bioanalyzer 2100 or gel electrophoresis. The labeled RNA was hybridized onto Arraystar Human small RNA Microarray (8 × 15 K, Arraystar, China). Besides, we scanned the array by an Agilent Scanner. The array images were construed by means of Agilent Feature Extraction software (version 11.0.1.1). GeneSpring GX v12.1 software package (Agilent Technologies) was applied to quantile normalization and follow-up data treating.

### Transwell assays

Cell migration assays were performed by determining the number of cells migrating across transwell chambers. We cultured transfected HaCat cells for 48 h, then seeded the HaCat cells in serum-free medium in the upper wells of the migration chambers (1 × 10^4^ cells/well). The lower wells contained the same medium with 10% serum. The plates were incubated for 24 h and the migrating HaCat cells were fixed with 70% ethanol. After staining with 0.25% crystal violet, the cells were counted using an inverted microscope (XD; Ningbo Sunny Instruments Co., Ltd., China).

### Western blotting

Cell lysis buffer (Beyotime Biotechnol, China) including 1 mM phenylmethyl-sulfonylfluoride (PMSF) was adopted, with the intention of treating the harvest cells for 20 min on ices. Centrifuge the cell lysates at 12,000 rpm for 15 min. A BCA kit (Cat# 23,225, Pierce, USA) was employed, with the intention of measuring the supernatant protein concentration. The protein was separated via sodium dodecyl sulfate–polyacrylamide gel electrophoresis (SDS-PAGE) and transferred with polyvinyl difluoride (PVDF) membranes. Five percent nonfat milk was applied, with the objective to blocking membranes at normal temperature for 2 h. Then, first antibodies of anti-PBX1 (1:1000, Ab97994, Abcam) and anti-GAPDH (1:1000, Ab9484, Abcam) were added to the membranes at the temperature of 4° during the night. We adopted TBS-T to rinse those membranes for three times. HRP-conjugated secondary antibody (1:2000, Ab205718, Abcam) was fostered using those membranes at normal temperature for 1 h. Protein bands were seen by means of one enhanced chemiluminescence (ECL) kit (Millipore, MA, USA). The protein band intensity was measured via ImageJ software. The internal reference was GAPDH.

### Dual‑luciferase report gene assay

QuickChange mutagenesis kit (Cat# 210515, Agilent Technologies, California, USA) was employed, with a view to constructing the 3ʹ-UTR fragment of PBX1 mRNA mutating. A broad-type 3ʹ-UTR fragment of PBX1 mRNA encompassing one putative miR-3074-5p-binding site (position 637–643) or mutation fragment was cloned into the psiCHECK2 vector, as PBX1-WT and PBX1-Mut. Lipofectamine 2000 was used for HaCaT cells transfection with psiCHECK2 vectors with PBX1-WT and PBX1-Mut. And 24 h later, they were transfected with miR-3074-5p inhibitor or mimics and their NC. Dual-luciferase reporter assay (DLRA) kit (Cat# 11402ES60, Yeasen, Shanghai, China) was adopted for the purpose of detecting the relative luciferase activity after transfection for 48 h. Renilla luciferase activity was measured and normalized to firefly luciferase.

### Statistical analysis

Experimental data is exhibited as the mean ± standard deviation (SD). Comparisons in multiple treatments were performed via one-way ANOVA or two-way ANOVA with Tukey’s post hoc test using GraphPad Prism version 7.03 (GraphPad Software Inc., Sandiego, CA, USA).

## Results

### Snora73 is significantly upregulated in psoriasis

**Fig. 1:**
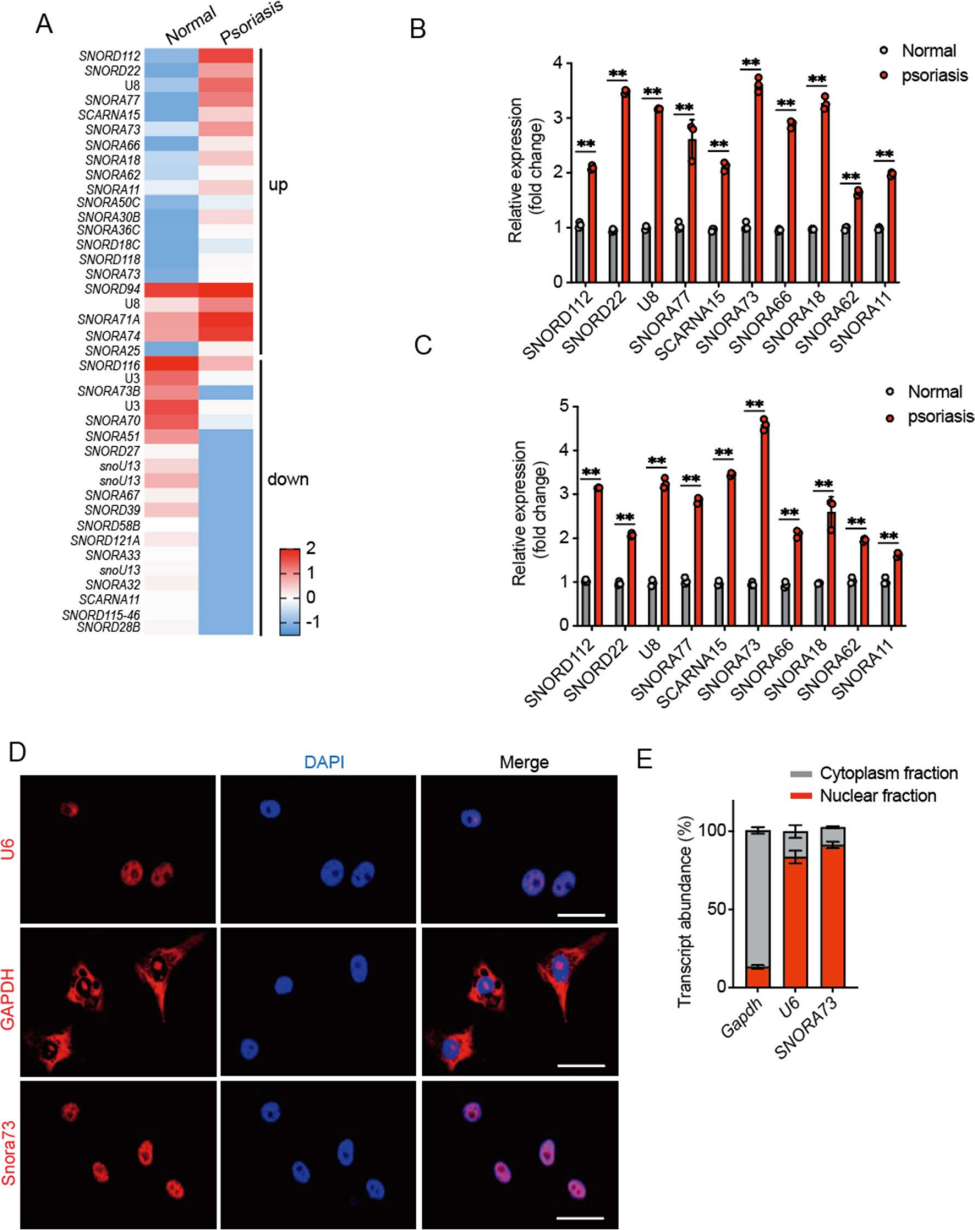
*Snora73* is highly expressed in psoriasis cells.** A** A heatmap of differentially expressed snoRNAs from normal and psoriasis samples. **B** Top ten upregulated snoRNAs mRNA expression in normal and psoriasis tissues were determined by qRT-PCR (*n* = 3). Loading control: 18S RNA. **C** Top ten upregulated snoRNAs mRNA expression were determined by qRT-PCR in normal (*n* = 3) and psoriasis (*n* = 3) blood samples. Loading control: 18S RNA. **D**, RNA FISH of *Snora73* in HaCat cells. Red (top) region represents *Snora73* distribution by antisense probe, the nuclei staining by DAPI is represented by the blue area. Scale bar, 50 µm. **E** *Snora73* cell distribution among HaCat cells measured via qRT-PCR (*n* = 3). U6 and GAPDH were markers in nuclear and cytoplasmic. **P < 0.01

To examine the functions of snoRNAs in psoriasis, the expression of snoRNAs transcripts in five matched pairs of psoriasis and normal tissues were characterized using RNA-seq analysis (Fig. 1A); we found that 23 upregulated snoRNAs (fold change > 1.5) and 19 downregulated snoRNAs (fold change < 0.7) were observed in psoriasis patients compared with normal subjects. And then, the expression of top 10 candidate snoRNAs was validated in psoriasis tissues and psoriasis plasma using qPCR; we observed that Snora73 was most upregulated in psoriasis tissues samples (Fig. 1B) and plasma samples (Fig. 1C) compared their normal control. FISH assay result revealed that Snora73 was mainly located in the nuclear (Fig. 1D), and these observations were further validated by nuclear-cytoplasmic separation assay (Fig. 1E). These findings reveal that Snora73 is highly expressed in psoriasis and may be a potential biomarker for the psoriasis.

### Snora73 promotes the progression of psoriasis

**Fig. 2:**
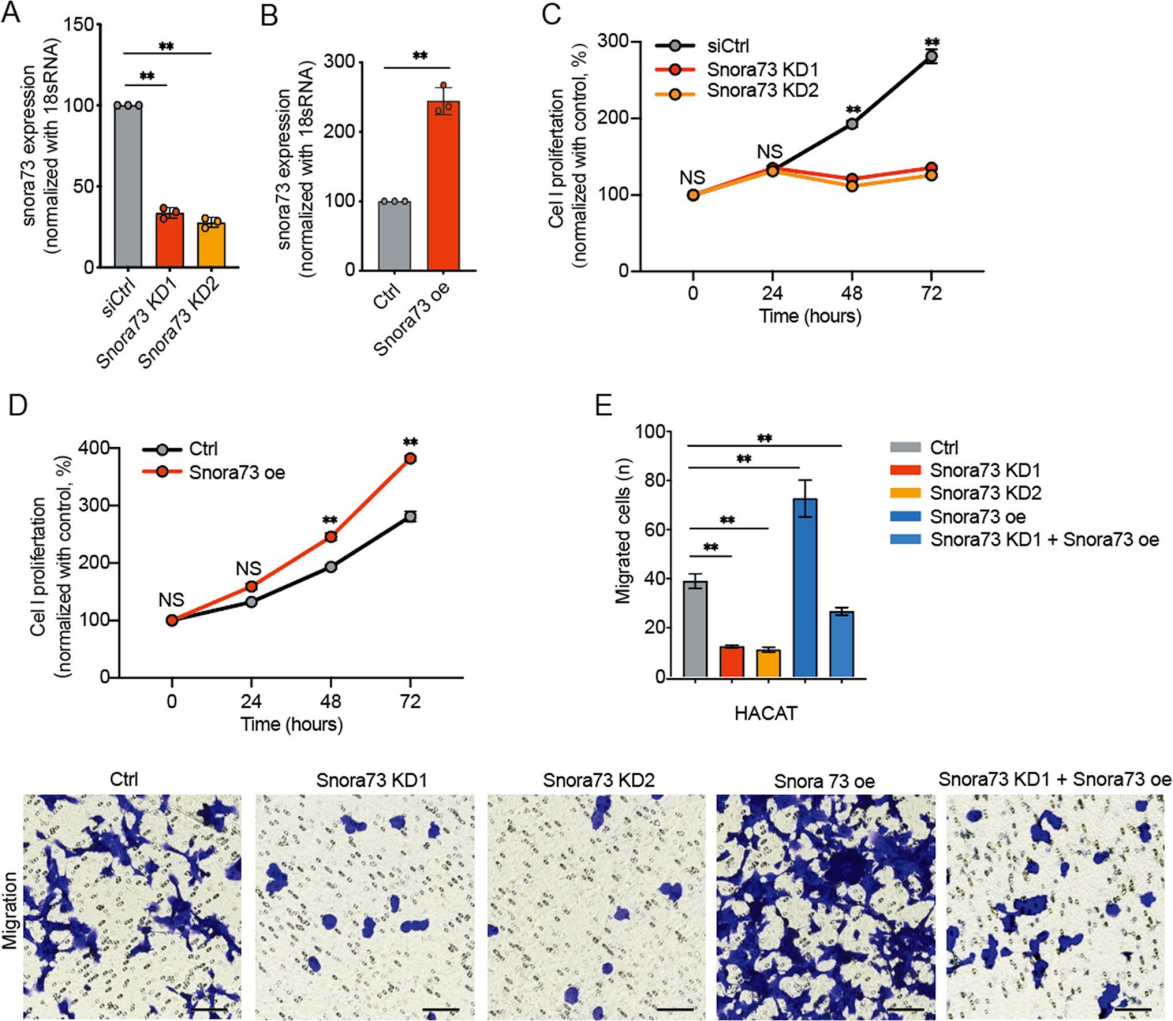
Snora73 promotes the progression of psoriasis.** A** qRT-PCR results of Snora73 expression in HaCaT cells with Snora73 knockdown (Snora73 KD #1 and Snora73 KD #1) (*n* = 3). **B** qRT-PCR results (*n* = 3) of Snora73 expression in HaCaT cells with Snora73 overexpression (Snora73 oe). **C**,** D** CCK-8 assay showed cell proliferation after Snora73 knockdown and Snora73 overexpression in HaCaT cells (*n* = 3). **E** The effect of overexpression (Snora73 oe) or knockdown (Snora73 KD1 and Snora73 KD2) of Snora73 on HaCaT cells migration were investigated using transwell assay (*n* = 3). *P < 0.05; **P < 0.01; ns, no difference

Two siRNAs targeting Snora73 and overexpression plasmids were transfected into HaCat cells to explore the functions of Snora73. QPCR results confirmed the efficiency of Snora73 knockdown and overexpression (Fig. 2A, B). We also observed that Snora73 knockdown could obviously suppress the cell proliferation in both Snora73 KD1 and KD2 HaCaT cells compared with control (Fig. 2C), while the Snora73 overexpression (Snora73 oe) exerted opposing effects (Fig. 2D). Moreover, cell migration was obviously decreased in Snora73 knockdown cells, and these abilities were rescued in Snora73 of HaCaT cells (Fig. 2E). These outcomes showed that Snora73 promotes the progression of psoriasis.

### Snora73 promotes psoriasis progression by sponging miR-3074-5p

**Fig. 3:**
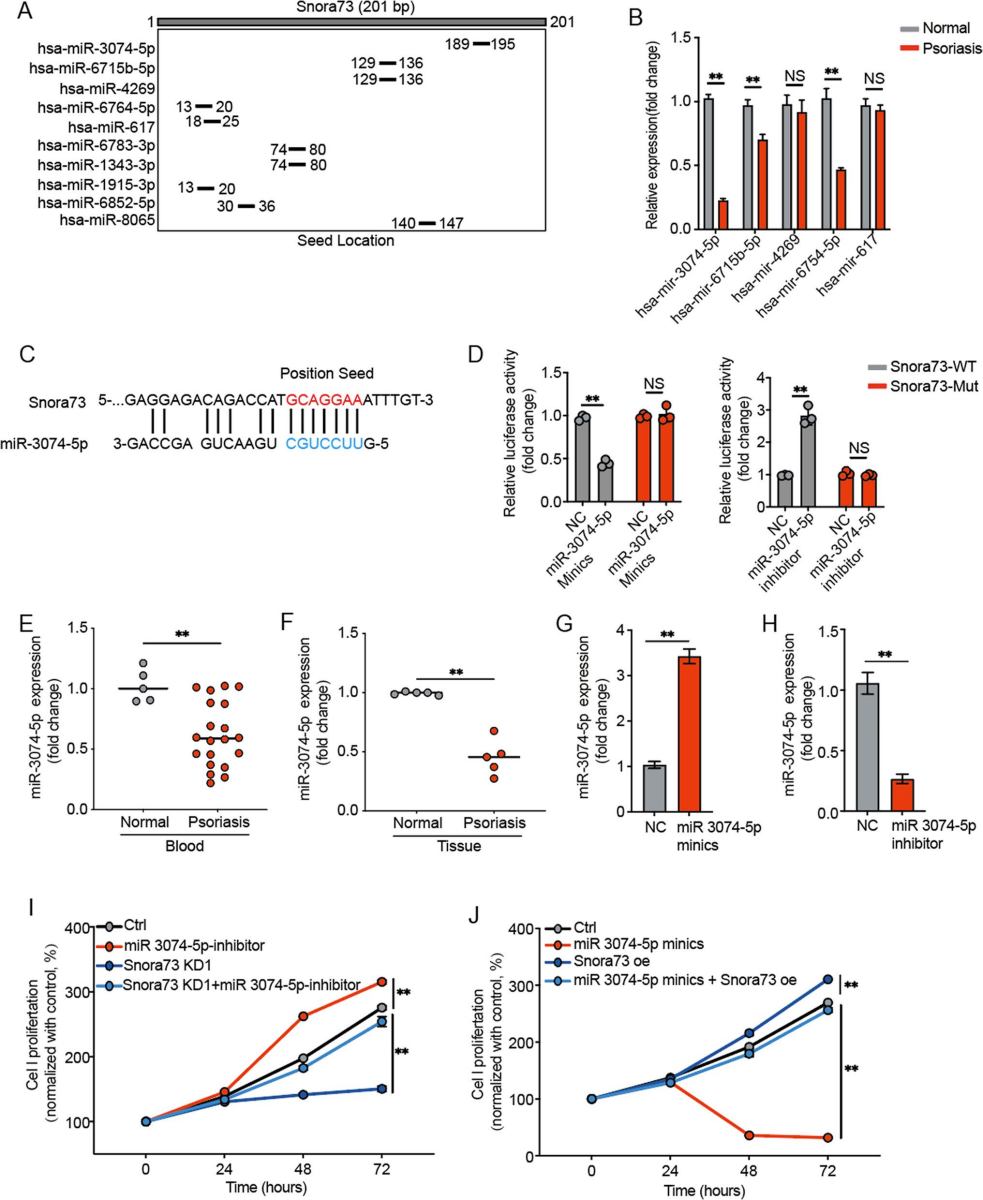
Snora73 promotes psoriasis progression by sponging miR-3074-5p. **A** The potential binding miRNAs of Snora73 predicted by miRTarBase, and 10 Snora73 candidate targets were list. **B** qRT-PCR results (*n* = 3) of top 5 Snora73 candidate targets (miR-3074-5p, miR-6715b-5p, miR-4269, miR-6754-5p, and miR-617) expression in normal and psoriasis tissues. Loading control: U6. **C** Schematic diagram of one predictive binding position of miR-3074-5p among Snora73 regions. **D** Dual luciferase reporter assays in HaCaT cells of miR-3074-5p binding sites predicted in Snora73 3′-UTR regions. **E**,** F** miR-3074-5p expression in tissues and psoriasis blood by qPCR (*n* = 3) compared with normal, U6 worked as one loading control (for blood samples: normal = 5, psoriasis = 20; for tissue samples: normal = 5, psoriasis = 5). **G**,** H** qRT-PCR (*n* = 3) was performed, with the aim of checking miR-3074-5p level among HaCaT cells treated with miR-3074-5p mimics and inhibitor. **I**,** J** CCK-8 assay results of cell viability after knockdown of miR-3074-5p (miR-3074-5p suppressor) and overexpression (miR-3074-5p mimics) in HaCaT cells (*n* = 3). **P < 0.01; ns, no difference

Snora73 contains binding sites of multiple miRNAs using bioinformatics analysis by miRTarBase (Fig. 3A), and the top 5 scores were selected for further study. The expression of these five miRNAs were observed in HaCat cells; we found that miR-3074-5p was the most enrichment in Snora73 (Fig. 3B). Based on the bioinformatics analysis from miRTarBase database, we showed that miR-3074-5p binding sites in sequence of Snora73 (Fig. 3C). To further confirm that Snora73 could directly binds to miR-3074-5p, dual-luciferase reporter assays (DLRAs) were conducted. Within miR-3074-5p mimic in Snora73-WT group, the activity of luciferase was lessened, while within miR-3074-5p inhibitor in Snora73-WT group was increased, but did not change in Snora73-Mut group (Fig. 3D). MiR-3074-5p levels in the plasma (normal = 5, psoriasis = 20) and tissue (normal = 5, psoriasis = 5) obtained from psoriasis patients and healthy subjects were measured using qPCR, our outcome illustrated that the downregulation of miR-3074-5p was prominent in psoriasis plasma (Fig. 3E) and tissue (Fig. 3F) samples compared to healthy samples. Furthermore, rescue experiments were performed to study whether Snora73 exerts its biological function by sponging miR-3074-5p. The efficiency of miR-3074-5p upregulation or downregulation was validated in the mimics (Fig. 3G) as well as miR-3074-5p inhibitor (Fig. 3H) HaCat cells using qRT-PCR. In note, we found that miR-3074-5p deficiency could obviously increase HaCaT cells proliferation (Fig. 3I). By contrast, miR-3074-5p mimics decreased HaCat cells proliferation of compared with control (Fig. 3J). These indicated that miR-3074-5p reversed the Snora73 ability of boosting the psoriasis advancement.

### miR-3074-5p directly binds to the 3′-UTR of PBX1 mRNA to suppress its expression

**Fig. 4:**
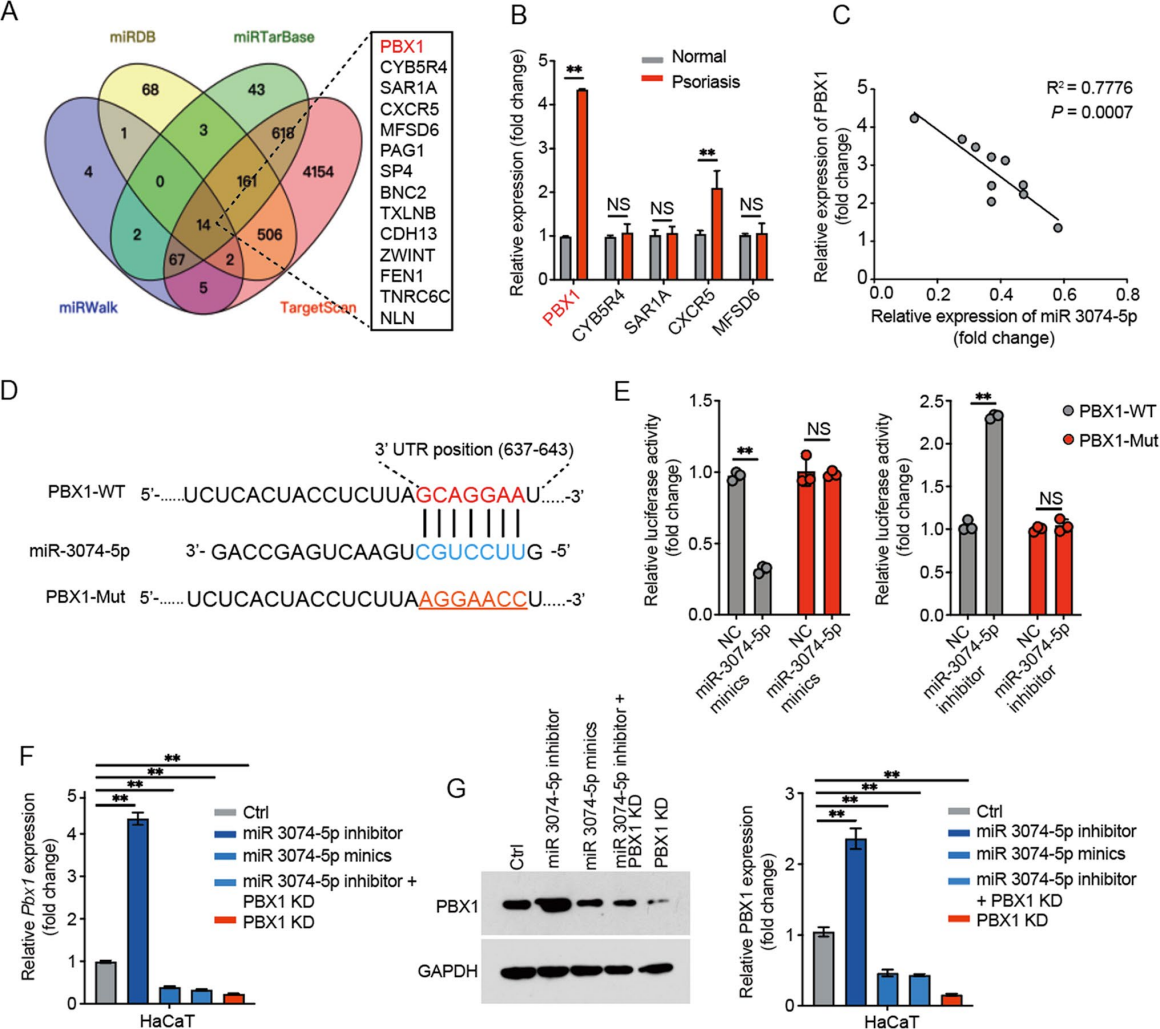
miR-3074-5p suppresses PBX1 expression through binding to its 3′-UTR.** A** Target mRNAs of miR-3074-5p forecast by miRTarBase, miRDB, TargetScan, and miWalk, and the 14 miR-3074-5p candidate targets were list on right. **B** qRT-PCR results (*n* = 3) of five miR-3074-5p candidate targets levels in psoriasis and normal tissues. **C** Pearson correlation analysis showed a passive association in ten psoriasis and ten normal tissues. **D** A predictive binding position of miR-3074-5p among PBX1 3′-UTR, the mutant sequence of binding positions is underlined. **E** Luciferase activity of luc-PBX1 were determined in HaCaT cells transfected with wild type of PBX1 (PBX1-WT)/mutant construct (PBX1-Mut) with miR-3074-5p inhibitor treatment or mimics (*n* = 3). **F** Relative *PBX1* mRNA levels in HaCaT cells was detected in multiple groups as indicated using qRT-PCR (*n* = 3). **G** The protein levels of *PBX1* was detected in multiple groups as indicated by Western blot (*n* = 3). **P < 0.01; ns, no difference

The miRTarBase (https://mirtarbase.cuhk.edu.cn/~miRTarBase/miRTarBase_2022/php/index.php), miRDB (https://mirdb.org/), TargetScan (https://www.targetscan.org/vert_80/), and miWalk (http://mirwalk.umm.uni-heidelberg.de/) databases were employed, with the aim of predicting the potential candidate genes of miR-3074-5p, and top 14 potential candidate genes (PBX1, CYB5R4, SAR1A, CXCR5, MFSD6, PAG1, SP4, BNC2, TXLNB, CDH13, ZWINT, FEN1, TNRC6C, and NLN) containing binding sites for miR-3074-5p were identified in all four databases (Fig. 4A). Then, the expression of top 5 potential candidate genes (PBX1, CYB5R4, SAR1A, CXCR5, MFSD6) of miR-3074-5p was detected in normal and psoriasis tissues by qPCR; our result revealed that PBX1 was the most upregulated genes in psoriasis tissues compare to normal ones (Fig. 4B). We also found that the PBX1 level was passively connected to the miR-3074-5p level among psoriasis tissues (Fig. 4C). To further verify the association between 3′UTR region of PBX1 and miR-3074-5p, we constructed a mutant reporter vector (PBX1-mut) with mutated binding position within the 3′UTR region (Fig. 4D). That luciferase activity does not have any change in co-transfection with the inhibitor or mimic of miR-3074-5p, as well as PBX1-mut (Fig. 4E). It was exhibited in rescued experiments that the inhibitor of miR-3074-5p raised the mRNA (Fig. 4F) and PBX1 protein (Fig. 4G) expression, whereas miR-3074-5p mimics had the antiphase effect. Furthermore, miR-3074-5p inhibitor also rescued the PBX1 mRNA and protein expression in PBX1 knockdown HaCaT cells (Fig. 4G). In conclusion, miR-3074-5p could negatively regulate PBX1 by binding to its 3′UTR.

### miR-3074-5p suppress psoriasis progression by inhibiting PBX1 expression

**Fig. 5:**
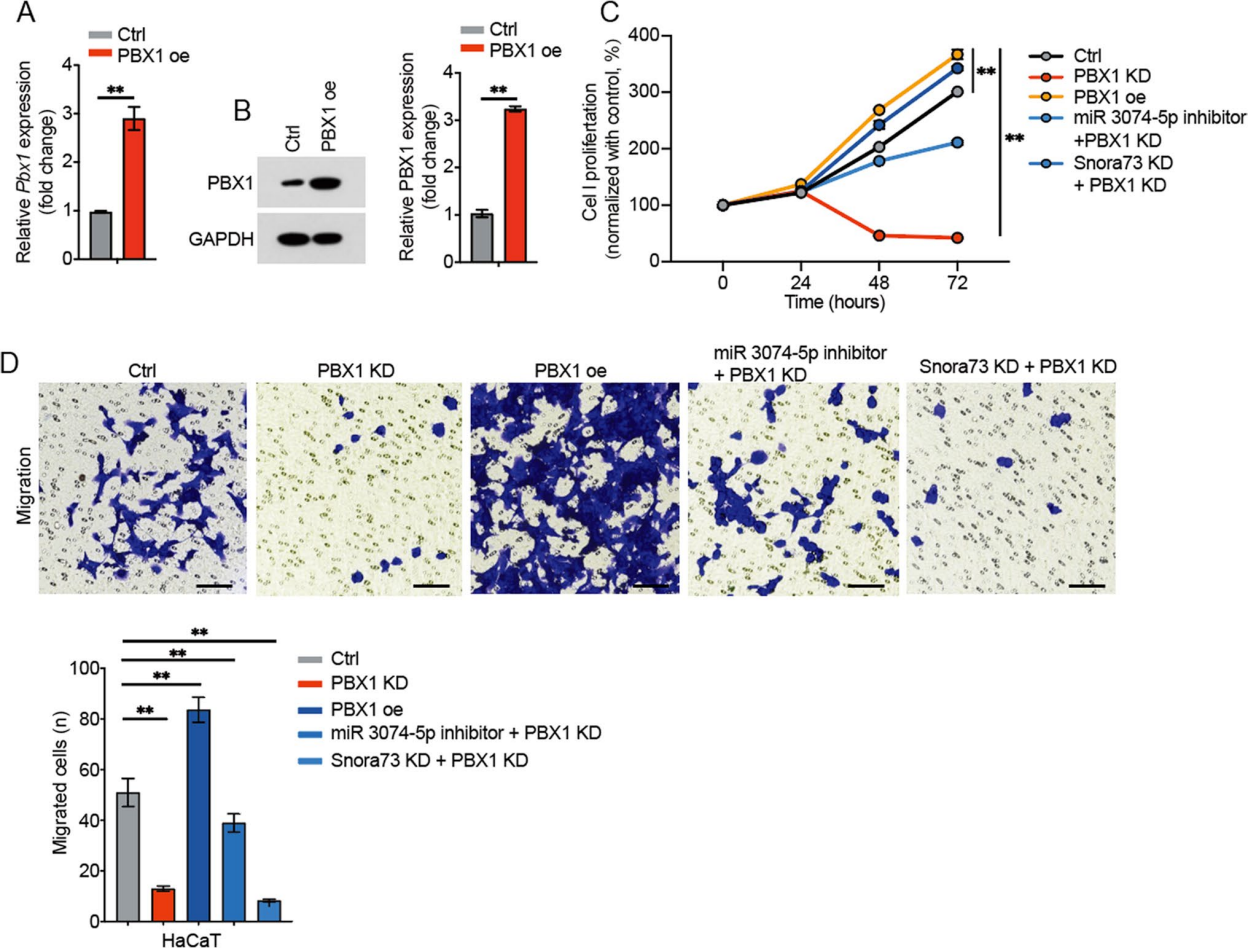
Snora73/miR-3074-5p axis regulates PBX1 expression.** A**,** B** PBX1 mRNA and levels of the protein among HaCaT cells which were transfected with overexpression plasmid via western blotting (*n* = 3) or qRT-PCR (*n* = 3). The cell proliferation was exhibited in **C,** CCK-8 assay after PBX1 knockdown (PBX1 KD) and overexpression (PBX1 oe) in HaCaT cells (*n* = 3). **D** The effect of overexpression (PBX1 oe) or knockdown (PBX1 KD) of PBX1 on psoriasis cell metastasis was investigated using transwell assay (*n* = 3). **P < 0.01

Next, we performed the PBX1 overexpression (PBX1 oe) HaCaT cells by Lentivirus, and then PBX1 overexpression was validated in mRNA (Fig. 5A) and the levels of protein (Fig. 5B) among PBX1 oe HaCaT cells compared with control. Functionally, PBX1 knockdown inhibited HaCaT cells proliferation (Fig. 5C) and migration (Fig. 5D), and PBX1 overexpression increased HaCaT cells proliferation and migration, while the miR-3074-5p inhibitor rescued these abilities. These suggest that miR-3074-5p inhibits psoriasis progression through inhibiting PBX1.

## Discussion

SnoRNAs are a kind of conserved nuclear RNAs with 60–300 nt and have significant roles in tumorigenesis because of ribonucleoprotein (RNP) guides in RNA modification (Zhou et al. 2017). SnoRNAs could be non-invasive biomarkers for diagnostics of malignancies due to its stably expressed and measurable in human bodies (Fitz et al. 2021; Nossent et al. 2019). Recently, many studies showed the potential role of snoRNAs in genetic disorders (Sahoo et al. 2008), human variation (Bhartiya et al. 2012), hematopoiesis (Bellodi et al. 2013), metabolism (Michel et al. 2011), and neoplasia (Mannoor et al. 2012). To our knowledge, there are no prior reports for snoRNA in psoriasis. Firstly, we screened the snoRNAs from five matched pairs of psoriasis and normal tissues using small RNA microarray assays and identified a novel and highly expressed Snora73 in psoriasis. We also showed that Snora73 was most highly expressed not only in psoriasis plasma but also in psoriatic lesions. Secondly, the role of Snora73 in psoriasis pathology was studied. We observed that Snora73 knockdown could obviously suppress psoriasis cell proliferation and migration, while the Snora73 overexpression exerted opposing effects. These findings reveal that Snora73 is highly expressed in psoriasis and promotes psoriasis progression, suggesting it may be a potential biomarker for the psoriasis.

MicroRNAs has crucial roles in psoriasis, such as keratinocyte hyperproliferation (Ghosh et al. 2023; Loganathan and Doss 2023; Xu et al. 2017; Yu et al. 2017), chemokine and cytokine production in keratinocytes (Shen et al. 2017), alongside mediation of immune disorder (Fu et al. 2015). Non-coding RNA modulated the miRNA activity through its miRNAs ‘‘sponges” function. However, how the regulatory mechanisms of miRNAs “sponges” by snoRNAs remains unclear. We found Snora73 contains binding sites of multiple miRNAs using bioinformatics analysis, and that miR-3074-5p was the most enrichment in Snora73 mRNA. The luciferase activity result confirmed that Snora73 could directly binds to miR-3074-5p. Furthermore, we showed the downregulation of miR-3074-5p among psoriasis tissues. The downregulation was passively linked to cell proliferation of psoriasis, indicating the inhibiting function of miR-3074-5p during the psoriasis progression.

Previous research has showed that pre-B cell leukemia homeobox-1 (PBX1) was involved in many important developmental programs, and its dysregulation associated with multifactorial disorders (Veiga et al. 2021). Though changing the regulatory miR-522-3p affinity to PBX1 3'-UTR, the rs6426881 T allele at PBX1 3′-UT is prominently correlated with breast carcinoma and gastric carcinoma (Mohammadi et al. 2021). Through targeting PBX1, microRNA-181 regulates posterior longitudinal ligament ossification (Liu et al. 2020). In our study, we found PBX1 3′-UTR contains binding sites of miR-3074-5p, which was confirmed using luciferase assay. MiR-3074-5p should conversely regulate PBX1 by binding to its 3′UTR. Functionally, PBX1 knockdown inhibited HaCaT cells proliferation and migration and PBX1 overexpression increased the HaCaT cell ability of proliferation and metastasis, while the miR-3074-5p inhibitor rescued these abilities. The limitation of the study is whether or how the effect of Snora73 in psoriasis progression in Snora73 knockdown mice model.

## Conclusion

In summary, we demonstrated that Snora73 was highly expressed in psoriasis, and Snora73 promoted the progression of psoriasis. Importantly, we found that Snora73 acted as a sponge for miR-3074-5p and PBX1 is a direct target of miR-3074-5p in psoriasis cells. This finding might constitute a potential therapeutic strategy in psoriasis.

### Supplementary Information

Below is the link to the electronic supplementary material.

Supplementary file1 (DOCX 22 KB)

## Data Availability

The dataset used and/or analyzed in this study is available from the corresponding author on reasonable request.
