# Correction to: Small nucleolar RNA Snora73 promotes psoriasis progression by sponging miR‑3074‑5p and regulating PBX1 expression

**DOI:** 10.1007/s10142-024-01306-1

**Published:** 2024-01-30

**Authors:** Lihua Zhang, Hui Guo, Xiaoguang Zhang, Ling Wang, Feng Wei, Yike Zhao, Bo Wang, Yibo Meng, Yanling Li

**Affiliations:** 1 Department of Dermatology, Clinical Medical Research Center of Dermatology and Venereal Disease in Hebei Province, The Second Hospital of Hebei Medical University, Shijiazhuang, 050000 China; 2 Construction Unit of the Sub‑Center of the National Center for Clinical Medical Research On Skin and Immunological Diseases, The Second Hospital of Hebei Medical University, Shijiazhuang, 050000 China; 3 Key Laboratory of Infection and Immunity of CAS, CAS Center for Excellence in Biomacromolecules, Institute of Biophysics, Chinese Academy of Sciences, Beijing, 100101 China

**Correction to: Functional & Integrative Genomics (2024)**

10.1007/s10142-024-01300-7

The original version of this article contains an error. Figure 2 and figure 5 was interchanged during online publication. This has been corrected now.

The original article has been corrected.

