# Supplementary material for: Small nucleolar RNA Snora73 promotes psoriasis progression by sponging miR-3074-5p and regulating PBX1 expression
Source: Funct Integr Genomics. 2024 Jan 19;24(1):15. doi: 10.1007/s10142-024-01300-7 (PMC10799104; doi:10.1007/s10142-024-01300-7)
Supplement: Supplementary file 1 — Supplementary file1 (DOCX 22 KB) [file 10142_2024_1300_MOESM1_ESM.docx]

**Supplementary Table 1. Baseline characteristics.**

|  | All patients (n=20) |
| --- | --- |
| Age, years | 45.1 (13.6) |
| Sex |  |
| Male | 14 (70%) |
| Female | 6 (30%) |
| PASI score | 20.1 (6.8) |
| IGA score |  |
| 3 (moderate) | 12 (60%) |
| 4 (severe) | 8 (40%) |
| DLQI score | 10.0 (6.8) |

Data are mean (SD) or n (%). PASI=Psoriasis Area and Severity Index. IGA=Investigator’s Global Assessment. DLQI=Dermatology Life Quality Index.

**Supplementary Table 2. qPCR primers used in this study**

| Genes | Forward (5’-3’) | | Reverse (5’-3’) |
| --- | --- | --- | --- |
| SNORD112 | | GGACCAATGATGAGACAGTGTT | ATGGACCTCAGTGTTTTGTGC |
| SNORD22 | | GTCCTAGTCCCAGAGCCTGTA | TCAGACAGTTCCTTCTGGAACAA |
| U8 | | TGGCCCACTTCTATGCCAAG | CAAGAAAGCTGGGGAGAGGG |
| SNORA77 | | ACTTCCAGGCAGGTGCTTTT | CATCGCCCTGGATAGGTGAG |
| SCARNA15 | | GCATGGCCGAATACTGTGTTT | AAGGGAAGACTGCTTTTGCAT |
| SNORA73 | | GCTCTGTCTAAATGGCATAGGG | TGTCCACAGGACTCAGAAGCT |
| SNORA66 | | ACTAGCTCTGCGTGATGTGG | CCTCAGTAGTGTCTGAGGCT |
| SNORA18 | | CACATCGTTGGAAACGCCTC | TGTCTTGTAATTCCTTCCCACAG |
| SNORA62 | | AGCTTGGAGTTGAGGCTACTG | TAGCGAAAACTTGCCCCTCA |
| SNORA11 | | CCCAAAGAATGGCTCCTCTGT | GAAGCCACAAGTTTGACGCC |
| 18SRNA | | TAACGAACGAGACTCTGGCAT | CGGACATCTAAGGGCATCACAG |
| GAPDH | | GCCGCATCTTCTTTTGCGTCGC | TCCCGTTCTCAGCCTTGACGGT |
| miR-3074-5p | | GCGGTTCCTGCTGAACTGA | AGTGCAGGGTCCGAGGTATT |
| miR-6715b-5p | | CAGGCACGACTGGTT | TGGTGTCGTGGAGTCG |
| miR-4269 | | GCAGGCACAGACAGCCCTG | GAACATGTCTGCGTATCTC |
| miR-6754-5p | | AACGAGACGACGACAGAC | CCAGGGAGGCTGGTTTGGAGGA |
| miR-617 | | CGTCCTCTGAAGGGTAAACTT | GCAGGGTCCGAGGTATTC |
| U6 | | CTGGTAGGGTGCTCGCTTCGGCAG | CAACTGGTGTCGTGGAGTCGGC |
| PBX1 | | CCCCCTTCCCTGTTTATCCTG | CAGCATGGGAATGCATCAGC |
| CYB5R4 | | GCATGGGTACAACCTGCTCT | GACAGTTGCACAAAGCAGCA |
| SAR1A | | TCTCATTGCCCAGTGGTGAC | ATACTGACCTGCACCAGCAC |
| CXCR5 | | GTGAGAGTGTCTTCACGGCA | TCCAGCCTCTTGCCTCTTTG |
| MFSD6 | | CTGCTCTTTGCCCTGATCCA | TGCGTGGCATGACATCTTCT |
